# Clinical Outcomes after Uncomplicated Cataract Surgery with Implantation of the Tecnis Toric Intraocular Lens

**DOI:** 10.1155/2016/3257217

**Published:** 2016-02-28

**Authors:** Wojciech Lubiński, Beata Kaźmierczak, Jolanta Gronkowska-Serafin, Karolina Podborączyńska-Jodko

**Affiliations:** Clinic of Ophthalmology, Pomeranian Medical University, 70-111 Szczecin, Poland

## Abstract

*Purpose*. To evaluate the clinical outcomes after uncomplicated cataract surgery with implantation of an aspheric toric intraocular lens (IOL) during a 6-month follow-up.* Methods*. Prospective study including 27 consecutive eyes of 18 patients (mean age: 66.1 ± 11.4 years) with a visually significant cataract and corneal astigmatism ≥ 0.75 D and undergoing uncomplicated cataract surgery with implantation of the Tecnis ZCT toric IOL (Abbott Medical Optics). Visual, refractive, and keratometric outcomes as well as IOL rotation were evaluated during a 6-month follow-up. At the end of the follow-up, patient satisfaction and perception of optical/visual disturbances were also evaluated using a subjective questionnaire.* Results.* At 6 months after surgery, mean LogMAR uncorrected (UDVA) and corrected distance visual acuity (CDVA) were 0.19 ± 0.12 and 0.14 ± 0.10, respectively. Postoperative UDVA of 20/40 or better was achieved in 92.6% of eyes. Mean refractive cylinder decreased significantly from −3.73 ± 1.96 to −1.42 ± 0.88 D (*p* < 0.001), while keratometric cylinder did not change significantly (*p* = 0.44). Mean absolute IOL rotation was 1.1 ± 2.4°, with values of more than 5° in only 2 eyes (6.9%). Mean patient satisfaction score was 9.70 ± 0.46, using a scale from 0 (not at all satisfied) to 10 (very satisfied). No postoperative optical/visual disturbances were reported.* Conclusion.* Cataract surgery with implantation of the Tecnis toric IOL is an effective method of refractive correction in eyes with corneal astigmatism due to the good IOL positional stability, providing high levels of patient's satisfaction.

## 1. Introduction

Approximately 60% of patients undergoing cataract surgery have more than 0.75 D of corneal astigmatism [1]. If uncorrected, this astigmatism results in reduced visual acuity and increased spectacle dependence in pseudophakic eyes [2]. The correction of corneal astigmatism in cataract surgery can be achieved using different surgical techniques (corneal or limbal relaxing incisions, modification of the placement of the incision site) [3, 4] or by implanting a toric intraocular lens (IOL) [5]. Several studies have reported successful visual and refractive outcomes after implantation of different models of toric IOL [5–19]. The Tecnis ZCT toric IOL combines an aspheric profile with a toric optic. To this date, only few clinical results with this specific type of toric IOL have been published, several with a rather short follow-up of less than 6 months [9, 13, 15, 16, 19]. The purpose of the current study was to report our clinical outcomes at 6 months after uncomplicated cataract surgery with implantation of the Tecnis ZCT toric IOL.

## 2. Methods

### 2.1. Patients

This nonrandomized prospective case series included 27 eyes of 18 patients undergoing cataract surgery with implantation of the Tecnis ZCT toric IOL (Abbott Medical Optics Inc.). Inclusion criteria were visually significant cataract, age of 18 years or older, and preoperative corneal astigmatism of 0.75 D or higher. Patients were excluded from the study when the following conditions were present: potential visual acuity of less than 0.2 LogMAR in each eye due to ocular pathological processes, systemic or ocular medication that could affect vision, any chronic or acute pathology that could alter the result, previous ocular surgery, amblyopia, strabismus, forme fruste or clinical keratoconus, pupil abnormalities, capsular or zonular abnormalities with the potential of inducing IOL decentration or tilting, and participation in another clinical study. The study adhered to the tenets of the Declaration of Helsinki and was approved by the local ethics committee.

### 2.2. Preoperative and Postoperative Evaluation

Before surgery, all patients underwent a complete ophthalmological examination that included the following: manifest refraction, measurement of LogMAR uncorrected (UDVA), and corrected distance visual acuity (CDVA), biometry and keratometry with the IOLMaster partial coherence interferometry device (Carl Zeiss Meditec AG), corneal topography to exclude irregular astigmatism, slit lamp examination, and dilated funduscopy. The IOL manufacturer's web-based toric calculator was used to determine the required cylinder power and axis for the IOL that was going to be implanted. The preferred clear corneal incision location was the superior temporal quadrant and the surgeon's estimated surgically induced corneal astigmatism was 0.75 D.

On the first day after surgery, the axis position of the implanted toric IOL was analyzed under pupil dilation (1.0% tropicamide) with the slit lamp by performing a thin coaxial slit rotation until it overlapped the axis margins on the IOL. In two cases of a rotation of the IOL axis of more than 5 degrees the IOL was repositioned in the operating room and were excluded from study. Six months after surgery, manifest refraction, LogMAR UDVA and CDVA, and corneal astigmatism were measured. Patients were asked about the incidence of postoperative optical/visual disturbances, such as arc of light, halos, ghosting, or glare, and about their satisfaction with the achieved visual outcome, using a scale from 0 to 10 (0 = not at all satisfied, 10 = very satisfied).

### 2.3. Intraocular Lens

The 1-piece aspheric toric IOL Tecnis ZCT has 6.0 mm optic diameter and an overall length of 13.0 mm. It has a 360-degree square edge with frosting to reduce migration of lens epithelial cells and possible glare effects. The C-loop haptics are aimed at providing a 3-point fixation in the capsular bag for maintaining good IOL centration and rotational stability. The lens is made of a hydrophobic acrylic material with a high Abbe value (55) which reduces the level of longitudinal chromatic aberration with the potential of improving contrast sensitivity [20].

### 2.4. Surgical Technique

Before surgery, after instilling topical anesthetic eye drops and with the patient in supine position, the corneal limbus was marked at the 0°, 90°, and 180° meridian using the toric reference marker AE 2791 (Asico). Intraoperatively the required IOL axis was determined with the aid of the axis marker AE 2794 (Asico). After phacoemulsification, the IOL was inserted into the capsular bag using the Unfolder Platinum 1 system (Abbott Medical Optics Inc.) through a 2.2 mm corneal incision in the superotemporal quadrant. After the removal of the ophthalmic viscosurgical device (Discovisc, Alcon) from the capsular bag, the IOL was rotated, if necessary, to the correct axis position.

### 2.5. Data Analysis

Distribution of analyzed data was performed using the Kolmogorov-Smirnov test. All data presented in the current study were not normally distributed and therefore nonparametric statistics were used. The Wilcoxon ranked sum test was used to compare changes in visual and refractive parameters between preoperative and postoperative examinations, considering a significance level of *p* < 0.05.

The spherocylindrical refractions obtained before and after surgery were converted to vectorial notation using the power vector method described by Thibos and Horner [21]. According to the power vector method, manifest refractions in conventional script notation (*S* [sphere], *C* [cylinder] ×  *φ*  [axis]) were converted to power vector coordinates and overall blurring strength (*B*) by the following formulas: *M* = *S* + *C*/2; *J*_0_ = (−*C*/2)cos⁡(2*φ*); *J*_45_ = (−*C*/2)sin⁡(2*φ*); and *B* = (*M* ^2^ + *J*_0_^2^ + *J*_45_^2^)^1/2^.

## 3. Results

Twenty seven eyes of 18 patients were enrolled in the study.  Table 1 summarizes the patient demographics and the preoperative data.

### 3.1. Visual and Refractive Outcomes

**Figure 1:**
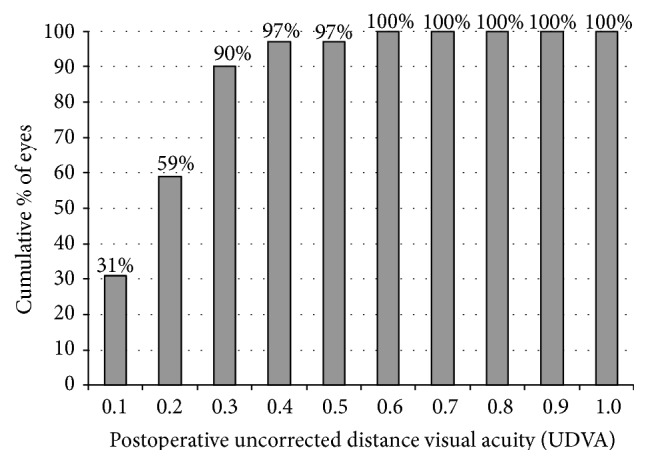
Distribution of the postoperative uncorrected distance visual acuity (UDVA) LogMAR.

**Figure 2:**
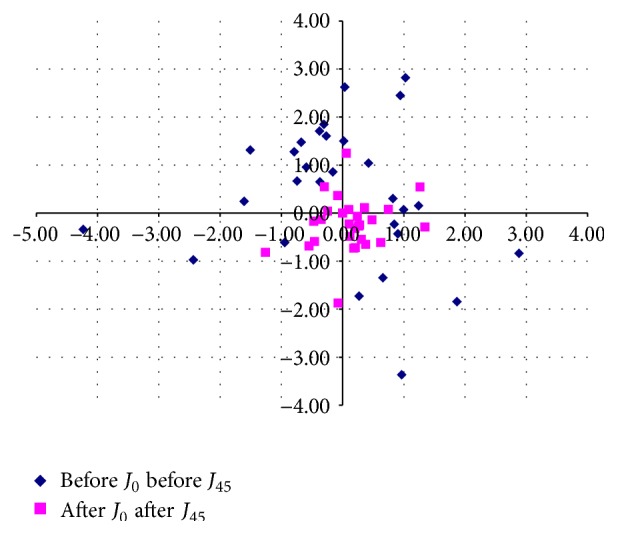
Distribution of the preoperative and postoperative *J*_0_ and *J*_45_ vectors representing the refractive astigmatism.

Table 2 shows the preoperative and 6-month postoperative visual and refractive outcomes in the analyzed sample. A significant improvement was found after surgery in LogMAR UDVA and CDVA (*p* < 0.001). At 6 months postoperatively, a LogMAR UDVA of 0.30 (20/40 Snellen) or better was achieved in 25 eyes (92.6%) (Figure 1). Postoperative CDVA was 0.30 or better (20/40 Snellen) in all eyes (100%) and 0.00 logMAR (20/20 Snellen) in 9 eyes (33.33%). All eyes (100%) showed a mean spherical equivalent within ±1.00 D of emmetropia. The refractive cylinder decreased significantly after surgery (*p* < 0.001) while the keratometric cylinder did not change significantly (*p* = 0.44).  Figure 2 shows the distribution of preoperative and postoperative *J*_0_ and *J*_45_ refractive cylinder vectors. Postoperatively, the data points concentrate around the origin (0,0) whereas preoperatively the data showed a high level of scattering.

### 3.2. Intraocular Lens Alignment

One day after surgery two eyes required additional repositioning surgery. Mean absolute IOL misalignment at 6 month after surgery was 1.1 ± 2.4 degrees (range, 0 to 8 degrees). Two eyes (7.4%) had an IOL misalignment of more than 5 degrees (8 degrees) and 4 eyes (17.2%) showed a misalignment of less than 5 degrees. Accurate alignment of the IOL with its intended axis was obtained in more than half of the operated eyes (19/27, 70.37%).

### 3.3. Patient Satisfaction and Visual Disturbances

At 6 months after surgery, mean patient satisfaction score was 9.70 ± 0.47 using a scale from 0 (not at all satisfied) to 10 (very satisfied). Specifically, 5 patients scored their satisfaction as 9 and the rest as 10. No optical or visual disturbances were reported by any of the patients from the analyzed sample.

### 3.4. Complications

There were no intraoperative complications. In one eye, a retinal detachment occurred at 2 months after surgery that was successfully treated by pars plana vitrectomy.

## 4. Discussion

Recently, the correction of corneal astigmatism in cataract surgery by implanting toric IOLs has gained popularity due to the increased patient demands and the excellent clinical outcomes reported with these IOLs [5]. In the current study we evaluated a specific modality of aspheric toric IOL, allowing the correction of a great variety of corneal astigmatism as it is available in cylinder powers of 1.00, 1.50, 2.25, 3.00, and 4.00 D (equivalent to 0.69, 1.03, 1.54, 2.06, and 2.74 D at the corneal plane, resp.). Some cases of higher corneal astigmatism which cannot be completely controlled with the available cylinder powers of this IOL model have nevertheless been included in our series. In general, good visual and refractive outcomes have been obtained with the Tecnis toric IOL, mainly due to its good positional stability.

Our results confirmed the results of previous studies evaluating the same IOL and demonstrating its ability as an effective method of corneal astigmatism reduction [9, 13, 15, 16, 19]. Specifically, we found a mean reduction in refractive astigmatism of 2.31 D that was statistically significant. At 6 months after surgery, refractive astigmatism ranged from 0.00 to −3.75 D, with a mean value of −1.42 ± 0.88 D. Lower postoperative refractive cylinder values have been reported by other authors evaluating the same type of toric IOL [9, 13, 15, 16, 19]. Waltz et al. [9] found a mean percentage of refractive cylinder reduction of 76.27 ± 33.09% at 6 months after cataract surgery with implantation of the Tecnis toric IOL, but in a group of eyes only requiring cylinder correction of 0.75 to 1.50 D. In our sample, twelve eyes (37.04%) had a preoperative corneal astigmatism of more than −3.00 D in which corneal astigmatism was not corrected completely but reduced significantly. Iovieno et al. [22] obtained a mean postoperative refractive cylinder of −1.81 ± 1.10 D in a group of eyes with high corneal astigmatism (preoperative refractive cylinder: −4.72 ± 1.13 D) undergoing cataract surgery with implantation of a custom-made high-power toric IOL. Cervantes-Coste et al. [23] found a residual refractive cylinder of 0.55 ± 0.60 D at 3 months after cataract surgery with implantation of a toric IOL in 19 eyes with symmetric corneal astigmatism of more than 2.25 D. Ouchi and Kinoshita [24] found similar results in another sample of eyes undergoing cataract surgery with implantation of a toric IOL for the correction of corneal astigmatism of more than 2.50 D (mean postoperative refractive cylinder: 1.07 ± 0.60 D). Some authors have even reported the necessity of implanting two IOLs in piggyback for achieving an acceptable refractive outcome in eyes with high corneal astigmatism [25]. The toric IOL evaluated in our sample was able to provide an effective correction of corneal astigmatism, even in cases requiring high levels of correction, reaching a mean percentage of refractive cylinder reduction of 61.93 ± 18.4%. All eyes (100%) had a mean postoperative spherical equivalent within ±1.00 D. This is consistent with the results of previous studies evaluating the same toric IOL [13] and also other modalities of toric IOLs [5, 11, 12].

In agreement with the good refractive outcomes, excellent UDVA results were obtained which was the main reason for the high levels of postoperative patient satisfaction. Mean LogMAR UDVA was 0.19 with all eyes achieving a value of 0.30 LogMAR or better, which is an outcome comparable or even better than that reported by other authors investigating toric IOLs [6–17, 26, 27]. Sheppard et al. [15] reported that 88% of eyes achieved a UDVA of 20/40 or better after implantation of the same toric IOL as evaluated in our series. Similarly, Ferreira and Almeida [16] found a postoperative UDVA of 0.3 logMAR or better in 100% of eyes implanted with the same toric IOL, whereas Mazzini [13] found that postoperative UDVA was 0.1 LogMAR (20/25) or better in 94.74% of eyes. Alió et al. [26] reported a postoperative UDVA of at least 20/40 in 76% of eyes implanted with a specific modality of microincision toric IOL. Kersey et al. [27] reported a mean postoperative UDVA value of 0.1 LogMAR in a sample of eyes implanted with a specific type of toric IOL, with 93% of eyes achieving a value of 0.3 LogMAR or better. LogMAR CDVA was also excellent in our series, with a mean postoperative value of 0.13.

Postoperative rotational stability of a toric IOL has a crucial influence on the final visual outcome. An undesirable postoperative IOL rotation may be the result of several factors, such as an incomplete removal of viscoelastics from the eye (reduced friction between the haptics and capsular bag with postoperative intraocular pressure changes) [28] or a postoperative significant capsule shrinkage. In the current study, mean IOL misalignment from the target axis was very small (1.1 ± 2.4°, range, 0 to 8°), which is consistent with the good visual and refractive outcomes obtained. Other studies evaluating the same IOL have reported similar or slightly higher levels of IOL misalignment [9, 13, 15, 16, 19]. Ferreira and Almeida [16] found a mean toric IOL axis misalignment of 3.15° in 20 eyes at 2 months after the implantation of the same toric IOL as used in our study, with no IOL rotating more than 10°. Similarly, Sheppard et al. [15] obtained 2 months postoperatively a mean IOL axis misalignment of 3.40° in a sample of 67 eyes, with only one eye showing a rotation of more than 10°. At 6 months after surgery, Mazzini [13] found a mean IOL misalignment of 3.33° in a sample of 19 consecutive eyes, with none of the eyes showing an IOL rotation of more than 7°. Hirnschall et al. [19] and Waltz et al. [9] obtained mean IOL misalignments of 3.6° and 1.89 ± 2.27° for the same toric IOL, respectively. Compared to other models of toric IOLs, our results were similar or better [6, 7, 10, 11, 14, 26, 27]. A mean IOL misalignment of 7.67 ± 4.04° was reported by Lam et al. [7] in a study evaluating a group of eyes implanted with a specific type of toric IOL. Miyake and coauthors [10] observed a mean IOL rotation of 4.5 ± 4.9° within 1 day postoperatively in a group of eyes implanted with a specific modality of aspheric toric IOL (Acrysof IQ toric SN6AT). These authors found that the rotation was more than 20° in 6 eyes, all of which had an axial length of more than 25.0 mm, with all rotations occurring within 10 days postoperatively [10]. Therefore, the aspheric toric IOL evaluated in the current study provided an excellent rotational stability which seemed to be mainly related to the IOL material (hydrophobic acrylic) [29] and design (3-point fixation system, offset haptics) [30].

Finally, patient satisfaction was high, with all patients scoring the visual outcome with a value of 9 or 10 in a scale from 0 to 10 postoperatively. Ahmed et al. [31] found in a group of patients implanted with a specific type of toric IOL that satisfaction with vision was rated 7 or higher by 94% of patients using a similar scale. These same authors found that the frequency and severity of halos and glare were significantly reduced pre- to postoperatively [31, 32]. In our sample, no postoperative optical/visual disturbances were reported by any patient, which is consistent with the optical quality outcomes reported by other authors with this type of toric IOL [13, 16].

In conclusion, cataract surgery with implantation of the Tecnis ZCT toric IOL provides an effective and predictable refractive correction in eyes with low to high levels of preexisting corneal astigmatism, providing high levels of visual quality and patient satisfaction. This might be due to the excellent rotational behavior of the IOL. Future studies should be conducted in order to evaluate the long term clinical outcomes with this modality of aspheric toric IOL.

## Figures and Tables

**Table 1:** Patient demographics and preoperative data in the analyzed sample.

Parameter	Value
Age (years)	
Mean ± SD	66.1 ± 11.4
Range	37 to 79
Sex (%)	
Male	6 (33%)
Female	12 (67%)
Sphere (D)	
Mean ± SD	−2.70 ± 6.70
Range	−18.50 to 5.50
Cylinder (D)	
Mean ± SD	−3.73 ± 1.96
Range	−8.50 to −1.50
Keratometry (D)	
K1 (steep)	42.58 ± 1.61
K2 (flat)	45.77 ± 1.82
Axial length (mm)	
Mean ± SD	23.87 ± 1.38
Range	22.19 to 27.83
Mean IOL power (D)	
Sphere	19.59 ± 4.58
Cylinder	−3.64 ± 0.54

**Table 2:** Preoperative and 6-month postoperative visual and refractive outcomes.

Parameter (mean ± SD)	Preoperative	6 months postoperative	*p* value
LogMAR UDVA	0.78 ± 0.22	0.19 ± 0.12	<0.001^*∗*^
LogMAR CDVA	0.49 ± 0.39	0.14 ± 0.10	<0.001^*∗*^
Manifest refraction			
Sphere (D)	−2.70 ± 6.70	0.62 ± 0.58	0.11^*∗*^
Cylinder (D)	−3.73 ± 1.96	−1.42 ± 0.88	<0.001^*∗*^
Spherical equivalent (D)	−4.63 ± 7.24	0.13 ± 0.43	0.01^*∗*^
Keratometric cylinder (D)	−3.19 ± 1.59	−3.16 ± 1.44	0.44

^*∗*^Value statistically significant.
